# M2 Macrophages Guide Periosteal Stromal Cell Recruitment and Initiate Bone Injury Regeneration

**DOI:** 10.3390/biomedicines12061205

**Published:** 2024-05-29

**Authors:** Dazhuang Lu, Yingfei Zhang, Shimin Liang, Yang Li, Jia Qing, Lanxin Gu, Xiuyun Xu, Zeying Wang, Xin Gao, Hao Liu, Xiao Zhang, Yongsheng Zhou, Ping Zhang

**Affiliations:** 1 Department of Prosthodontics, Peking University School and Hospital of Stomatology, 22 Zhongguancun South Avenue, Haidian District, Beijing 100081, China; ludazhuang666@gmail.com (D.L.); zyfdentist@163.com (Y.Z.); shimin.liang@foxmail.com (S.L.); 15896260251@163.com (Y.L.); bjmuqingjia@bjmu.edu.cn (J.Q.); green306@163.com (L.G.); xvxiuyun123@163.com (X.X.); wangzeying723@hotmail.com (Z.W.); gaox0724@163.com (X.G.); kqliuhao@bjmu.edu.cn (H.L.); kqzhouysh@hsc.pku.edu.cn (Y.Z.); 2 National Center for Stomatology, National Clinical Research Center for Oral Diseases, National Engineering Research Center of Oral Biomaterials and Digital Medical Devices, 22 Zhongguancun South Avenue, Haidian District, Beijing 100081, China; 3 Beijing Key Laboratory of Digital Stomatology, National Health Commission Key Laboratory of Digital Technology of Stomatology, 22 Zhongguancun South Avenue, Haidian District, Beijing 100081, China; 4 Institute of Advanced Clinical Medicine, Peking University, No.38 Xueyuan Road, Haidian District, Beijing 100191, China

**Keywords:** mesenchymal stromal cells (MSCs), periosteal stromal cells (PSCs), craniofacial injury, macrophages (MØs), Neuregulin 1 (NRG1), single-cell sequencing

## Abstract

The periosteum plays a critical role in bone repair and is significantly influenced by the surrounding immune microenvironment. In this study, we employed 10× single-cell RNA sequencing to create a detailed cellular atlas of the swine cranial periosteum, highlighting the cellular dynamics and interactions essential for cranial bone injury repair. We noted that such injuries lead to an increase in M2 macrophages, which are key in modulating the periosteum’s immune response and driving the bone regeneration process. These macrophages actively recruit periosteal stromal cells (PSCs) by secreting Neuregulin 1 (NRG1), a crucial factor in initiating bone regeneration. This recruitment process emphasizes the critical role of PSCs in effective bone repair, positioning them as primary targets for therapeutic interventions. Our results indicate that enhancing the interaction between M2 macrophages and PSCs could significantly improve the outcomes of treatments aimed at cranial bone repair and regeneration.

## 1. Introduction

The craniomaxillofacial region is among the most complex areas of the human body, containing multiple cavities and intricate bone structures. Developmental anomalies, injuries, and tumors can significantly complicate clinical bone reconstruction in this area [1]. Clinical trials have validated stem cell-mediated bone regeneration for craniomaxillofacial defects. Notably, mesenchymal stromal cells (MSCs) are favored due to their ethical acceptability, ease of acquisition, robust proliferation, and multi-lineage differentiation capabilities [1]. Current research primarily focuses on the identity and biological functions of characterized bone marrow stromal cells (BMSCs), such as Gremlin 1-expressing cells (Grem 1^+^) [2], skeletal stromal cells (SSCs) expressing leptin receptor (LEPR^+^) [3], Nes^+^ cells [4], and COL2A1^+^ cells [5]. However, due to the significant anatomical, developmental, and ossification differences between long bones and craniofacial bones, these cells may not fully represent the stem cells in craniofacial tissues [6,7,8].

Unlike long bones, which contain expansive bone marrow cavities, craniofacial bones are often thin and dense, resulting in the cortical bone comprising most of the craniofacial bone volume [9]. Moreover, the periosteal tissue covering cranial and maxillofacial bones has garnered increased attention. Beyond basic functions such as nourishment, protection, and sensation, periosteal tissue also possesses osteogenic capabilities [10], directly participating in bone formation through the secretion of paracrine factors [11,12], leading to new layered bone formation in response to changes in mechanical forces [13]. For example, femurs covered with periosteum exhibit higher bone strength than those without [14]. Additionally, the role of periosteal tissue in response to bone injury is significant, with studies showing that the inflammatory response in the periosteum can effectively activate the migration and proliferation of MSCs post-injury [15,16,17]. Following bone injury, periosteal activation (PA) and thickening begin within the first 24 h [15], accompanied by a proliferative response from periosteal stromal cells (PSCs) [18]. Moreover, the cell content in the periosteal callus even correlates with the extent of the injury [19,20].

Although the existing studies on periosteal-derived stem cells (PDSCs) have shed light on some PDSCs considering their identities and functions in bone reconstruction [21,22,23,24,25,26,27], these studies were conducted using rodent models, which differ significantly from human structures in terms of development and anatomy and thus impede clinical translation. In this study, we aimed to utilize Bama miniature pigs as animal models to investigate PSCs with varying characteristics in the cranial periosteum, employing single-cell sequencing technology to explore the functional relationship between macrophages (MØs) and PSCs following craniofacial bone injury.

## 2. Materials and Methods

### 2.1. Extraction of Cranial PSCs from Miniature Pigs

Animal anesthesia was administered via the intramuscular injection of 1% isopentobarbital (g/g, USP, Rockville, MD, USA), followed by the routine preparation of the operative area. A T-shaped incision was made at the junction between the posterior edge of the ears and the median sagittal cranial line. Skin and subcutaneous tissues were incised straight to the bone surface. The flap was opened evenly from the midline to the upper edge of the orbital part on both sides. Subcutaneous tissues on the bone surface were removed using sharp surgical instruments, and the entire skull was incised from front to back, following the naso-frontal suture, across the orbital floor to the back of the occipital bone, with the brain tissue being discarded. The medial and lateral periostea of the skull bone were carefully peeled off, with the periosteal collection focused on the bone sutures. When separating the periosteal tissue post-injury, the separation of the medial and lateral periostea began at least 2 mm from the suture mesenchymal region to preserve the suture periosteum for subsequent examination. Tissues were then immediately placed in a buffer solution (8% FBS, 2% penicillin and streptomycin, 90% PBS).

Type II and Type IV collagenase (0.2%, Sigma-Aldrich, St. Louis, MO, USA) were added after thoroughly mincing the tissue, and the mixture was shaken for 40 min (80–100 rpm). The tissue cell suspension was filtered through a gradient filter (100 µm and 70 µm) and then placed in ice-cold PBS buffer to stop digestion. Cells were collected after centrifugation, and a brief treatment with ice-cold sterile water was conducted to dissociate blood cells [28,29]. The cell suspension was then resuspended in fresh PBS to obtain a cell suspension derived from periosteal tissue. Cells were cultured in a proliferation medium (PM), including α-minimum essential medium (α-MEM) (Gibco, Grand Island, CA, USA), 10% (*v*/*v*) fetal bovine serum (FBS) (Gibco, Grand Island, CA, USA), and 1% penicillin/streptomycin (Gibco, Grand Island, CA, USA). The adherent cells obtained were identified as PSCs.

### 2.2. Extraction of Monocytes from Miniature Pigs

An appropriate volume (10–15 mL) of peripheral blood from the miniature pigs was centrifuged at 1800 rpm for 10 min. We then discarded the supernatant and retained the cell precipitate, which was washed 3 times with PBS, carefully discarding the supernatant each time. The final cell precipitate was then resuspended and transferred into a Petri dish.

### 2.3. Collection of M2-Type Macrophage-Derived Medium (M2DM)

For the M2DM collection, monocytes extracted from the miniature pigs’ peripheral blood were cultured in the previously mentioned proliferation medium (PM), supplemented with M-CSF (50 ng/mL, MCE, Princeton, NJ, USA). The medium was changed every 2 days, and non-adherent cells were removed to isolate macrophages. The monocytes/macrophages were then induced with IL-4 (40 ng/mL) for 24 h to achieve M2 polarization. The medium was subsequently replaced every 3 days and centrifuged at 5000 rpm, and the M2DM was collected. Modified M2DM for subsequent tests involved mixing the M2DM with cell proliferation medium (PM) and osteogenic induction medium (OM) in a 1:1 ratio [30].

### 2.4. Critical Cranial Bone Defect Model in Miniature Pigs

The animal anesthesia and surgical procedures were similar to those previously described. After fully exposing the operative area along the T-shaped incision, the periosteum on the external side of the bone wall was carefully released using a sharp periosteal stripper to expose the bone surface. Utilizing a 5 mm diameter circumferential cutting drill (Vincent Medical, Hong Kong, China), a circular bone defect was meticulously created on both sides of the median sagittal sutures, at least 5 mm away from the median suture area, just anterior to the occipito–parietal suture, ensuring the preservation of the internal periosteum. Subsequently, the periosteum and skin tissue were sutured together, facilitating successful animal recovery.

To investigate the function of the PSCs across the periosteal anatomical regions after cranial bone injury, the periosteal tissue was separated from the medial and lateral bone plates, maintaining a minimum distance of 2 mm from the median suture area to preserve the suture periosteum for future experiments.

### 2.5. Healing Model of Skull Defect in C57BL/6 Mice

To evaluate the osteogenic capacity of periosteal cells in situ during the defect-healing process, a critical cranial bone defect model was established in male C57BL/6 mice. The procedure began with skin preparation and sterilization. An approximately 2 cm incision was made atop the cranial plate following anesthesia, revealing the entire skull bone. The subcutaneous tissue was carefully separated along the median sagittal suture. At the junction of the parietal and interparietal bones on the median sagittal suture, a precise 2 mm diameter bone defect was created, with special care taken to preserve the medial periosteum and protect the brain. Cells were then incorporated into Hyaluronic Acid Methacryloyl (HAMA, EFL Tech, Suzhou, China), as per the guidelines. Approximately 5 × 10^5^ cells in a 20 µL volume were precisely deposited to fill the bone defect completely and were then cured with UV light for enhanced stabilization. Following the surgery, animals were revived with standard postoperative care.

### 2.6. Cell Culture and Cell Differentiation Detection

Human bone marrow-derived mesenchymal stem cells (hBMMSCs) were sourced from Scien-Cell (San Diego, CA, USA), and periosteum cells from miniature pigs were derived from tissue dissociation and enzymatic digestion in this study. Cells were cultured in a PM, containing α-minimum essential medium (α-MEM) (Gibco, Grand Island, CA, USA), 10% (*v/v*) fetal bovine serum (FBS, Gibco, Grand Island, CA, USA), and 1% penicillin/streptomycin (Gibco, Grand Island, CA, USA). All cells were incubated at 37 °C with 5% CO_2_ for growth. Differentiation induction commenced when the confluence reached 70% or more.

Osteogenic differentiation was induced using an osteogenic medium (OM) comprising the PM, dexamethasone (10 nM), vitamin C (200 μM), and β-glycerophosphate (10 mM) (all from Sigma-Aldrich, St. Louis, MO, USA). Both media were refreshed every 3 days for subsequent testing.

### 2.7. Fluorescence-Activated Cell Sorting (FACS) for Macrophages

Periosteal cell suspension from the miniature pigs was adjusted to a 5 mL volume at a concentration of 1 × 10^6^ to 10^7^ cells/mL using PBS buffer, after red blood cells were removed, and was maintained at 4 °C for the FACS preparation.

CD11B antibody (Cat#101207, Biolegend, San Diego, CA, USA) conjugated with PE and CD206 antibody (Cat#321103, Biolegend, San Diego, CA, USA) conjugated with FITC were used for sorting and analyzing M2-type macrophages. Cell suspensions from the periosteum were mixed with primary antibodies at a ratio of 1 µL antibody per 100 µL (*v*/*v*) and incubated in the dark at 4 °C for 1 h. Cells underwent 3 washes with PBS buffer to remove unbound antibodies and were then resuspended and analyzed through flow cytometry on a BD SymphonyS6 System.

### 2.8. ALP Staining and Quantitative Analysis

A 12-well cell culture plate was used for cell culturing for 7 days using the PM or OM. Cells were washed 3 times with PBS (1 min each). After removing the residual solution, cells were fixed with 95% (*v*/*v*) ethanol at room temperature (RT) for 30 min. Staining was performed using a kit from the Jiancheng Bioengineering Institute, strictly following the manufacturer’s instructions. After staining, cells were thoroughly washed with clean water and then air-dried at room temperature before microscopic observation. Quantitative analysis of the ALP activity was conducted using an ALP activity kit from the Jiancheng Bioengineering Institute, China, with the signals normalized based on the protein content.

### 2.9. ARS Staining and Quantitative Analysis

To evaluate the osteogenic differentiation and mineralization after 21 days of induction, Alizarin Red S (ARS) staining was employed. Initially, cells were fixed with 95% ethanol for 20 min and were then thoroughly washed with distilled water. Cells were stained with a 1% Alizarin Red S solution for 10 min, followed by multiple washes with distilled water to remove excess dye, and were then air-dried for the microscopic observation of the mineralized nodules. For the quantitative analysis of the mineralization, mineral deposits were dissolved in a 10% cetylpyridinium chloride (CPC) solution. The absorbance was measured at 490 nm using a microplate reader.

### 2.10. Colony Formation Test

To assess the colony-forming unit (CFU) formation, cells were seeded at a density of 1000 cells per well in a 12-well plate. The culture medium was refreshed every other day to maintain optimal growth conditions. After 1 week, cells were fixed with 4% paraformaldehyde at room temperature for 20 min to preserve the cell structure. Post-fixation, cells were stained with 1% crystal violet for 20 min to enhance the colony visibility. The stained colonies were subsequently counted and recorded for each well.

### 2.11. Cell Proliferation Detection by CCK8

Cells were plated at a density of 5000 per well in a 96-well plate and incubated for 24 h to ensure adequate attachment. At designated intervals (1, 3, 5, and 7 days), 10 µL of CCK-8 reagent was added to each well and the plates were incubated at 37 °C for 2 h to develop color. Absorbance was measured at 450 nm using a microplate reader. The absorbance from the blank wells was subtracted from that of the sample wells to calculate the cell viability and proliferation rates.

### 2.12. Dorsal Subcutaneous Cell Transplantation in Nude Mice

To assess the in vivo osteogenic potential of periosteal cells, dorsal subcutaneous cell transplantation was performed. A minimum of 100,000 cells were used per transplantation site. These cells were centrifuged, collected, and uniformly mixed with β-TCP scaffold materials (0.05 g per site) from REBONE Biomaterials (Shanghai, China), and they were then incubated at 37 °C for 40 min to ensure uniformity. Under complete anesthesia, longitudinal incisions were made on the dorsal surfaces of BALB/c female nude mice, with the careful separation of the bilateral skin and mucosa. The prepared cell-β-TCP suspension was then deposited into the interspaces created between the skin flaps and fascia. The incisions were meticulously sutured closed. The sites were left to heal for 6 weeks, after which the transplanted tissues were harvested for further analyses.

### 2.13. Micro-Computed Tomography (Micro-CT)

Mouse skull-healing tests were conducted using a high-resolution Inveon Micro-Computed Tomographer (Siemens, Munich, Germany). Scanning parameters were set at 60 kV, 220 μA, and a resolution of 8.0 μm, with exposure times ranging from 8 to 15 min, resulting in a pixel size of 8.6 × 8.6 × 8.6 μm^3^. Three-dimensional images were captured and reconstructed. Quantitative analysis was performed using Inveon Research Workplace software (ver. 3.3.0, Siemens, Germany).

The imaging analysis focused on new bone formation within 1 mm of the bone defect sites. The quantified parameters included the bone mineral density (BMD) (g/cm^3^), bone volume-to-total volume ratio (BV/TV) (%), trabecular thickness (Tb.Th) (mm), trabecular number (Tb.N) (1/mm), and trabecular separation (Tb.Sp) (mm), using the software of CT Analyser (ver. 1.20.8, Bruker, Leipzig, Germany).

### 2.14. Scratch Wound-Healing Assay

Cells were seeded in a 12-well plate and cultured until they reached full confluence. The medium containing 10% FBS was then replaced with a medium containing 1% FBS for a 24 h starvation period prior to the assay. Using a 1 mL pipette tip, guided by a ruler, uniform and straight scratches were made in the center of each well. The monolayer was washed with serum-free medium to remove detached cells. The wound closure was monitored, and images were captured every 6 h. Image J software (version 3.1) was used to measure and analyze the remaining scratch area, with a smaller remaining area indicating a higher cell migration ability.

### 2.15. Detection of Immunohistochemistry (IHC)

Periosteum tissues were extracted and prepared as frozen sections prior to IHC detections. Tissue sections were initially incubated with the primary antibodies Anti-PRRX1 (Cat#ab211292, Abcam, Cambridge, UK), Anti-CD11B (Cat#ab133357, Abcam), and Anti-CD206 (Cat#ab313398, Abcam) for 1 h at 4 °C. Subsequently, they were incubated with Alexa Fluor^®^ 647-conjugated Goat Anti-Mouse and Alexa Fluor^®^ 488-conjugated Goat Anti-Rabbit secondary antibodies (Cat#ab150115 and ab150077, Abcam). After staining with DAPI for nuclear visualization, the sections were mounted with Fluoromount-G. Finally, the sections were imaged using the Vectra Polaris™ system.

### 2.16. Single-Cell Sequencing Analysis

Approximately 20,000 cells were initially processed, with around 10,000 ultimately captured for analysis. A cDNA library was prepared using the Chromium Single-Cell 3.0 Kit and was sequenced on the Illumina NovaSeq platform. Following the sequence acquisition, the data were aligned with the mm10 reference genome of the mouse. Using Cellranger software (ver. 7.1.0, 10× Genomics, Pleasanton, TX, USA), Unique Molecular Identifiers (UMIs) were collapsed to produce a gene expression matrix at the single-cell level. This matrix was analyzed using the Seurat software package (ver.4.1.0, Satija Lab, New York, NY, USA). Within this framework, genes active in fewer than three cells were excluded, as were cells with over 25% mitochondrial gene expression or fewer than 300 genes. Seurat’s FindVariableGenes function identified genes with variable expressions across cells, and principal component analysis (PCA) was used to reduce the dimensionality. The t-SNE method was then applied for visualization based on the PCA results. Cell types within different subclusters were annotated based on the expressions of characteristic genes.

### 2.17. Statistical Analyses

In this study, all values are presented as means ± standard deviations (SDs). Statistical analysis was conducted using SPSS software (ver. 23.0; IBM Corp, Armonk, NY, USA), and data representation was performed with GraphPad Prism software (ver. 23.0; GraphPad Software, Inc., San Diego, CA, USA). Independent two-tailed Student’s *t*-tests were used to analyze the differences between the two groups, while a one-way Analysis of Variance (ANOVA) followed by Tukey’s post hoc test or the Least Significant Difference (L.S.D) test were employed for multiple group comparisons. Statistical significance was established at a *p*-value of less than 0.05, balancing Type I and Type II errors to ensure the reliable detection of the true differences among the groups.

## 3. Results

### 3.1. Cranial Bone Injury Enhanced the Osteogenic Activity of PSCs

**Figure 1:**
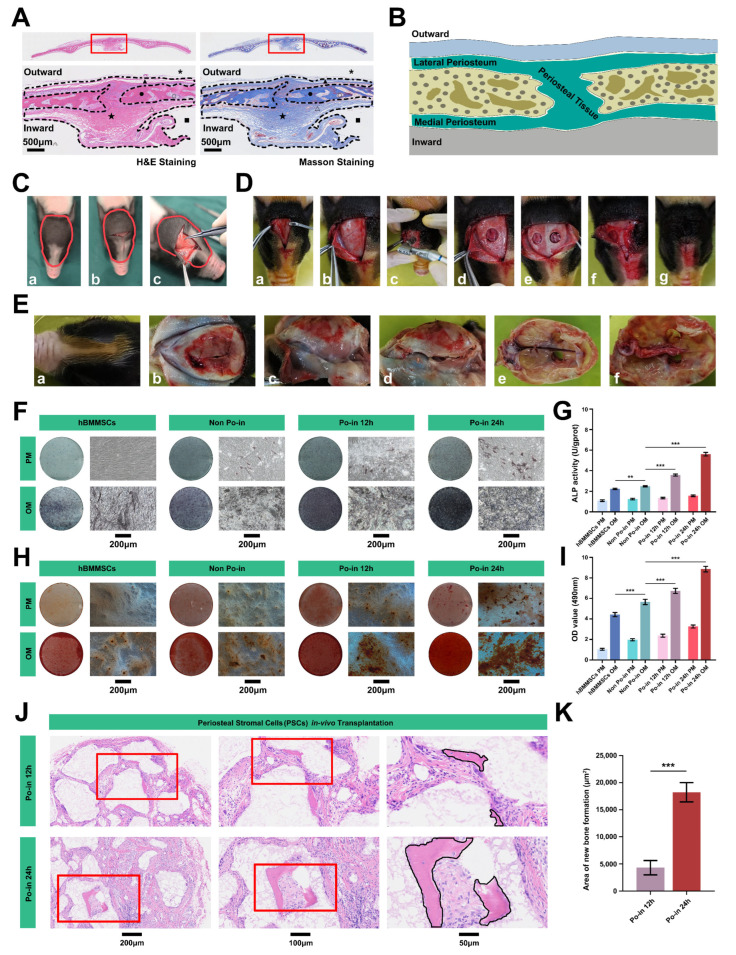
Enhanced osteogenic activity of PSCs following cranial bone injury. (**A**) H&E and Masson staining of cranial bones, periostea, and marrow cavities in neonatal miniature pigs. The region enclosed by the black dotted line represents the periosteal tissues. *, skin and muscle tissue (removed); ▲ and △, periosteal tissues on the lateral and medial cranial bone plates; ● and ○, bone trabecula and bone marrow cavities in cranial bone plate; ■, brain tissue (removed); ★, cranial sutural mesenchymal tissue. Scale bar: 500 μm. (**B**) Diagram illustrating the structure of the cranial bone and periosteum. The area in indigo represents the cranial periosteum tissue. (**C**) The intraoperative approach for obtaining cranial periosteal tissue from a miniature pig. (**a**–**c**), surgical area preparation, intraoperative incision and periosteal tissue indication. The region enclosed by the red line represents the sampling area. (**D**) Surgical process for constructing a cranial bone injury model in miniature pigs. (**a**), incision and flap open; (**b**). periosteal tissue free; (**c**–**e**). bilateral skull defect modeling; (**f**,**g**), periosteum and flap sutured separately. (**E**) Procedure for preparing and extracting cranial periosteal tissue in the bone injury model of miniature pigs. (**a**,**b**), operative area preparation and wound open; (**c**–**e**), disassemble the entire skull following the anatomical markers; (**f**), peel the entire periosteum for cell extraction. (**F**) ALP staining demonstrating the in vitro osteogenic capacity of hBMMSCs and PSCs before and after cranial bone injury. PM: proliferation medium; OM: osteogenic medium; scale bar: 200 μm. (**G**) Quantitative analysis of ALP activity from (**F**) (*n* = 3, **, *p* < 0.01, ***, *p* < 0.001). (**H**) ARS staining illustrating the in vitro osteogenic capacity of hBMMSCs and PSCs before and after cranial bone injury. PM: proliferation medium; OM: osteogenic medium; scale bar: 200 μm. (**I**) Quantitative analysis of ARS staining from (**H**) (*n* = 3, ***, *p* < 0.001). (**J**) H&E staining of in vivo subcutaneous PSC graft specimens. Red squares represent the local zoom area. Areas outlined by the black line indicate new bone formation (scale bars: 200, 100, and 50 μm). (**K**) Quantitative analysis of new bone formation areas from (**J**) (*n* = 3, ***, *p* < 0.001).

To investigate PSCs in the cranial vaults of neonatal minipigs, our study focused on a specific region encompassing the periosteal tissues of the frontal, parietal, and interparietal bones (Figure 1A). We meticulously removed the periosteal mesenchymal tissue from both the medial and lateral sides of the cranial bony plates and from the bony sutures (Figure 1A–C), isolating whole PSCs through mechanical shearing and enzymatic digestion. Following brief saline rinses of the tissue (three times), erythrocytes were eliminated from the cell suspension via rapid lysis in sterile water [28,29]. Concurrently, to assess the biological function of the PSCs before and after injury, we inflicted bilateral perforations (5 mm diameter) on the bony plates (Figure 1D) and collected the PSC suspension after 12 and 24 h (Po-in 12 h and Po-in 24 h), alongside a non-injury control group (Non Po-in) for comparation (Figure 1E).

We separated the PSC suspension into two fractions for the osteogenic induction culture and conducted ALP and ARS staining at 7 days and 21 days of induction, respectively. We found that, compared to the human bone marrow mesenchymal stem cells (hBMMSCs), the non-injured minipig PSCs (Non Po-in) exhibited stronger osteogenic differentiation activity and mineralized nodule formation. More notably, these biological functions were further enhanced after cranial injury (Po-in 12 h and 24 h), and they continued to increase over time post-injury (Figure 1F–I). Subsequently, to precisely delineate the specific osteogenic function, the injured PSCs (Po-in 12 h and Po-in 24 h) were combined with β-TCP scaffold materials and transplanted subcutaneously onto the backs of nude mice. After six weeks, the transplants showed new bone formation in vivo, with the Po-in 24 h group exhibiting a stronger osteoblastic capability than the Po-in 12 h group (Figure 1J,K).

Therefore, we have substantial reasons to believe that cranial bone injury stimulation significantly enhances the osteogenic activity of PSCs in miniature pigs both in vivo and in vitro.

### 3.2. The Injury Reversed the Difference in the Osteogenic Ability of the PSCs in Different Regions

**Figure 2:**
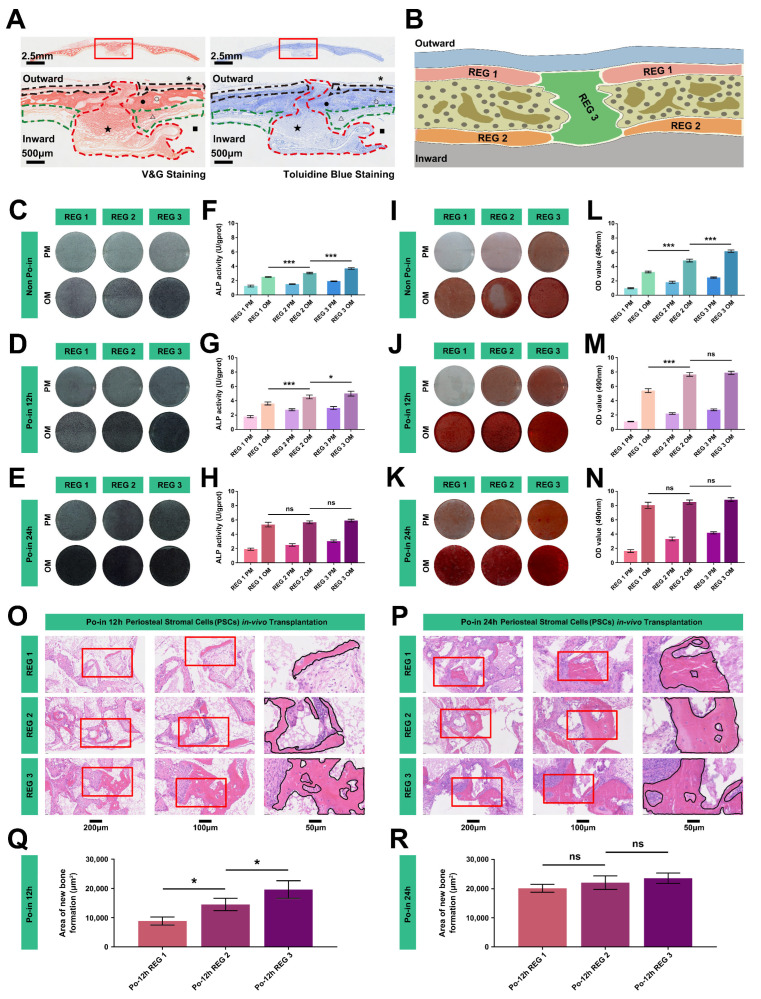
Reversal of osteogenic capability differences in PSCs from various regions post-injury. (**A**) Van Gieson (V&G) and Toluidine Blue staining of cranial bones, periostea, and marrow cavities in neonatal miniature pigs. The outlined areas represented by black, green, and red dotted lines indicate different spatial samples within the periosteal tissues. *, skin and muscle tissue (removed); ▲ and △, periosteal tissues on the lateral and medial cranial bone plates; ● and ○, bone trabecula and bone marrow cavities in the cranial bone plate; ■, brain tissue (removed); ★, cranial sutural mesenchymal tissue. Scale bar: 500 μm. (**B**) Diagram showing three target regions (REGs) of periosteal tissues. REG 1, periosteal tissue outside the cranial bone plate; REG 2, periosteal tissue inside the cranial bone plate; REG 3, periosteal tissue in the cranial bone suture. (**C**–**E**) ALP staining demonstrating the in vitro osteogenic ability of PSCs from the three periosteal REGs under various injury conditions. (**F**–**H**) Quantitative analysis of ALP activity from (**C**–**E**) (*n* = 3, *, *p* < 0.05, ***, *p* < 0.001, ns, non-significance). (**I**–**K**) ARS staining illustrating the in vitro osteogenic ability of PSCs from the three periosteal REGs under different injury conditions. (**L**–**N**) Quantitative analysis of ARS activity from (**I**–**K**) (*n* = 3, ***, *p* < 0.001). (**O**,**P**) H&E staining of in vivo subcutaneous PSC graft specimens from the three periosteal REGs under varying injury conditions. Red squares represent the local zoom area. Areas outlined by the black line indicate the formation of new bone. (**Q**,**R**) Quantitative analysis of new bone formation areas from (**O**,**P**) (*n* = 3, *, *p* < 0.05, ns, non-significance).

To explore the varied biological functions of PSCs after cranial injury and elucidate their behavior based on the spatial positions of bone sutures and periosteal tissue, we divided the periosteal tissue into three study regions (REGs 1–3) once again (Figure 2A,B). Initially, we examined the osteogenic activity of the PSCs from three different periosteal regions of the neonatal, non-injured miniature pigs (Non Po-in). As anticipated, the PSCs from the three anatomical REGs demonstrated distinct osteogenic activities and functions, as indicated by the ALP and ARS staining, with the cells derived from the cranial sutures exhibiting the strongest osteogenic activity and function among the three groups (Figure 2C–N).

We then assessed the osteogenic activity of the PSCs under post-injury conditions (12 h and 24 h) using the same detection methods for the three REGs. To our surprise, the osteogenic function of the PSCs from the three REGs not only strengthened over time post-injury but also became uniform after 24 h (Figure 2C–N). To confirm these findings, we conducted subcutaneous transplantation in nude mice, mixing the PSCs with β-TCP materials. After six weeks, H&E staining revealed that the differences in the osteogenic activity among the three REG-derived PSCs had disappeared following 24 h of injury (Figure 2O–R).

Despite the initial distinct osteogenic functional activities of the PSCs marked by temporal and spatial variations, these differences eventually homogenized post-injury. This suggests that PSCs may undergo temporal and spatial cell migration and relocation among different REGs.

### 3.3. Characterization of Periosteal Tissue Single-Cell Atlas

**Figure 3:**
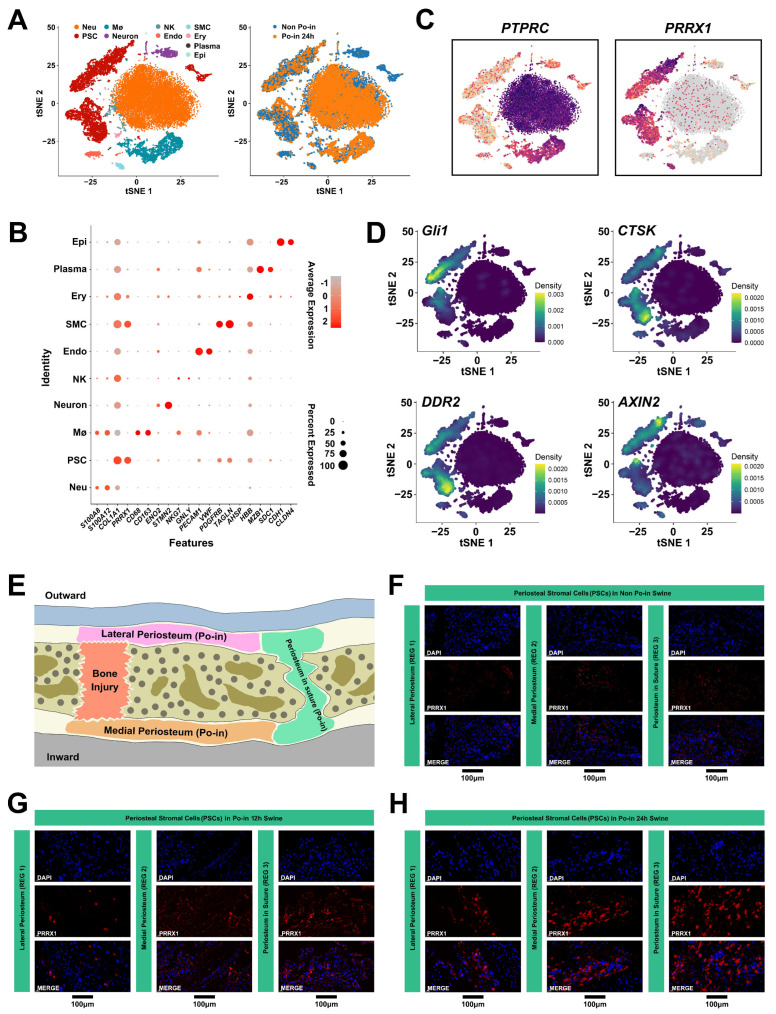
Characterization of the periosteal tissue single-cell atlas. (**A**) t-SNE visualization displaying cell cluster information of periosteal tissue under various cranial injury conditions (sequenced using the 10× Genomics technique) and directed cell source clusters. (**B**) Dot plot illustrating the expression patterns of representative cluster marker genes from (**A**). The color gradient from light gray to bright red indicates expression levels from low to high, while the dot size represents the percentage of cells expressing a specific gene. (**C**) Feature plot illustrating the expression levels of *PTPRC* and *PRRX1*. (**D**) t-SNE visualization showing the expression levels of classical periosteal stem cell markers (*GLI1*, *CTSK*, *DDR2*, and *AXIN2*). (**E**) Diagram indicating the locations of three examination areas in periosteal tissues under cranial injury (analogous to three REGs). (**F**–**H**). IHC clearly displaying the locations of the PSCs (marked by PRRX1) within three periosteal REGs under distinctive injury conditions (DAPI, nucleus; Red, PRRX1). Scale bar: 100 μm.

To delineate the cellular components of the periosteal tissue and examine changes in the PSCs post-injury, we conducted single-cell transcriptomic profiling using 10× Genomics scRNA-seq on fresh periosteal cells. After quality control, cells were obtained from the cranial periosteal tissues of both non-injured (Non Po-in) and post-injury (Po-in 24 h) minipigs (Figure 3A). The analysis identified 10 cell clusters, with batch effect correction in Harmony and unsupervised clustering in Seurat (Figure 3A). These clusters primarily included immune and stromal cells, identified by their representative marker genes: epithelial cells (expressing *CDH1* and *CLDN4*); plasm cells (expressing *MZB1* and *SDC1*); erythroblasts (expressing *HBB* and *AHSP*); smooth muscle cells (expressing *PDGFRB* and *TAGLN*); endothelial cells (expressing *VWF* and *PECAM1*); NK cells (expressing *GNLY* and *NKG7*); neurons (expressing *STMN2* and *ENO2*); macrophages (MØs) (expressing *CD68* and *CD163*); mesenchymal stem cells (expressing *PRRX1* and *COL1A1*); neutrophils (expressing *S100A12* and *S100A8*) (Figure 3B). The prevalent expressions of *PTPRC* and *PRRX1* indicated a composition predominantly consisting of immune and mesenchymal cells (Figure 3C). We mapped the classical periosteal stem cell surface markers—GLI1 [25], CTSK [10], DDR2 [27], and AXIN2 [26]—to these populations to clarify the distribution of the craniomaxillofacial tissue-derived stem cells in the miniature pigs, ultimately selecting PRRX1 as the representative marker for subsequent studies (Figure 3D).

To elucidate the conditions of the PSCs at different stages (before and after injury) and their specific periosteal locations, we performed immunohistochemistry (IHC) on the periosteal tissue, including PSCs from the aforementioned three regions (REGs), under skull injury conditions (Figure 3E). We observed that, although the PSCs (marked by PRRX1) were scattered throughout the periosteal tissues before injury (Non Po-in), they were predominantly concentrated in the cranial bone suture space (REG 3), followed by the medial periosteal tissue (REG 2), with minimal presence in the lateral periosteum (REG 1) (Figure 3F). However, the number of PRRX1-labeled PSCs in all three REGs increased over time post-injury (Po-in 12 h and 24 h), a trend that was particularly notable in REG 1, accompanied by elevated fluorescence expression levels of PRRX1 from 12 to 24 h post-injury (Figure 3G,H). This indicates that the skull injury stimulated an increase in the PSC numbers across all three REGs.

Thus, the results suggest a significant expansion and positional relocation (i.e., cell migration) of critical PSCs following cranial injury stimulation.

### 3.4. Cell–Cell Interaction Identification and Analysis of MØs in Cranial Injury

**Figure 4:**
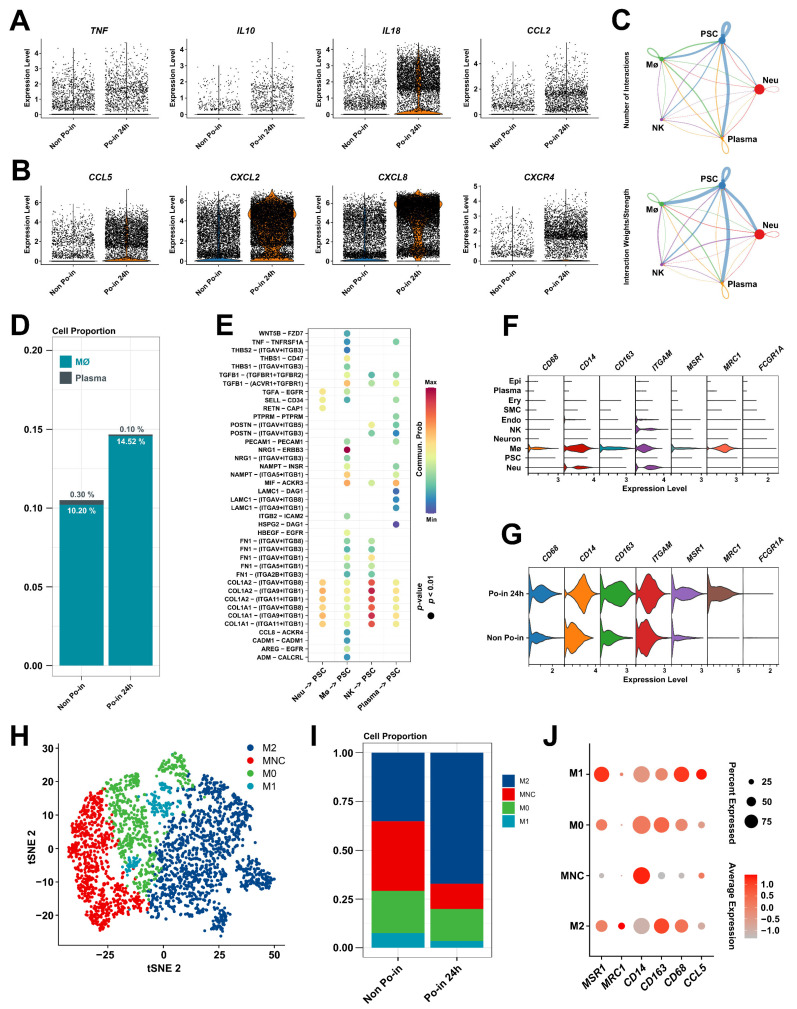
Cell–cell interaction identification and analysis in the cranial injury microenvironment. (**A**,**B**) Violin chart showing changes in IFs before and after injury. The X-axis represents the periosteal cell information; the Y-axis represents the expression levels. Horizontal dispersion indicates cell numbers. (**C**) Network diagram of cell–cell interactions among different cell clusters from cranial periosteal tissues. The size of the nodes represents the number of interactions, and the thickness of the lines indicates the strength of the interactions between linked clusters. (**D**) The changes in the cell proportions of macrophages and plasma cells before and after cranial bone injury. (**E**) Visualization of key ligand–receptor pairs between two selected cell–cell interaction clusters. The X-axis represents the two selected cell clusters; the Y-axis represents the ligand acceptor information. The size of the nodes represents the *p*-value, and the color gradient from indigo to red indicates the likelihood of communication. (**F**) Violin chart showing the enrichment of macrophage-related genes in cell clusters. The X-axis represents the gene expression levels; the Y-axis represents the information of each cluster. Vertical dispersion indicates cell numbers. (**G**) Violin chart depicting the enrichment of M2-related genes in periosteal tissues before and after cranial bone injury. The X-axis represents the gene expression levels; the Y-axis represents the information of each sample. Vertical dispersion indicates cell numbers. (**H**) Subpopulation analysis of MØs before and after cranial injury. (**I**) The changes in the proportions of macrophage subpopulations before and after cranial injury. (**J**) Dotplot illustrating the expression patterns of representative cluster marker genes of MØ subpopulations.

To investigate changes in the stem cell microenvironment before and after cranial injury, we initially examined the genes of inflammatory factors (IFs) that exhibited significant changes in the cell samples (Non Po-in and Po-in 24 h) through single-cell analysis. Compared to the normal condition (Non Po-in), cranial injury significantly increased the presence of inflammatory factors and chemokines, such as *TNF*, *IL10*, *IL18*, *CCL2*, *CCL5*, *CXCL2*, *CXCL8*, *CXCR4*, etc. (Figure 4A,B), suggesting that the immune function may influence the differentiation and functional activity of PSCs.

To understand the functional distinctions of PSCs after cranial injury, we investigated the interactions between PSCs and immune cells. Using the CellChat package [31], we identified close cellular interactions between PSCs and both MØs and plasma cells (Figure 4C). Cell proportion analysis before and after injury revealed a clear increase in MØs post-injury (Figure 4D). To further elucidate the regulatory mechanisms of MØs on PSCs, we highlighted the key cytokines that facilitate MØ-to-PSC crosstalk (Figure 4E). Notably, the ligand–receptor pairs involving NRG1 and ERBB3 were prominent in the interactions between the macrophages and PSCs. Other strong ligand–receptor interactions included TGFB1, NAMPT, and MIF with PSCs. Additionally, we observed that the injured group at 24 h showed higher expressions of M2 macrophage-related marker genes, such as *ITGAN*, *MSR1*, and *MRC1*, compared to the Non Po-in group (Figure 4F,G). A subpopulation analysis of the MØs revealed four subclusters: M0, M1, and M2 macrophages and monocytes (MNCs) (Figure 4H). Interestingly, the post-injury changes in these MØ subclusters were significant: the proportion of M2-type MØs (with high expression levels of *MRC1*) increased dramatically from about 30% to about 70%, while the number of M1-type MØs (expressing *CCL5*) was significantly reduced, accounting for only a small percentage (about 3%) (Figure 4I,J).

Based on this analysis, the substantial functional differences in PSCs before and after skull injury are likely related to the role of macrophages in the immune response, particularly the M2-type MØs.

### 3.5. MØs Are Crucial in the Initial Stages of Bone Reconstruction after Cranial Injury

Based on the above results, we confirmed that PSCs serve as stem cells and found that MØs are likely involved in the biological regulation of PSCs after cranial injury. To explore the potential regulatory role of MØs, we examined both PSCs (PRRX1) and MØs in cranial periosteal tissue at various stages before and after injury (CD11B for macrophages and CD206 for the M2 subtype [32]).

**Figure 5:**
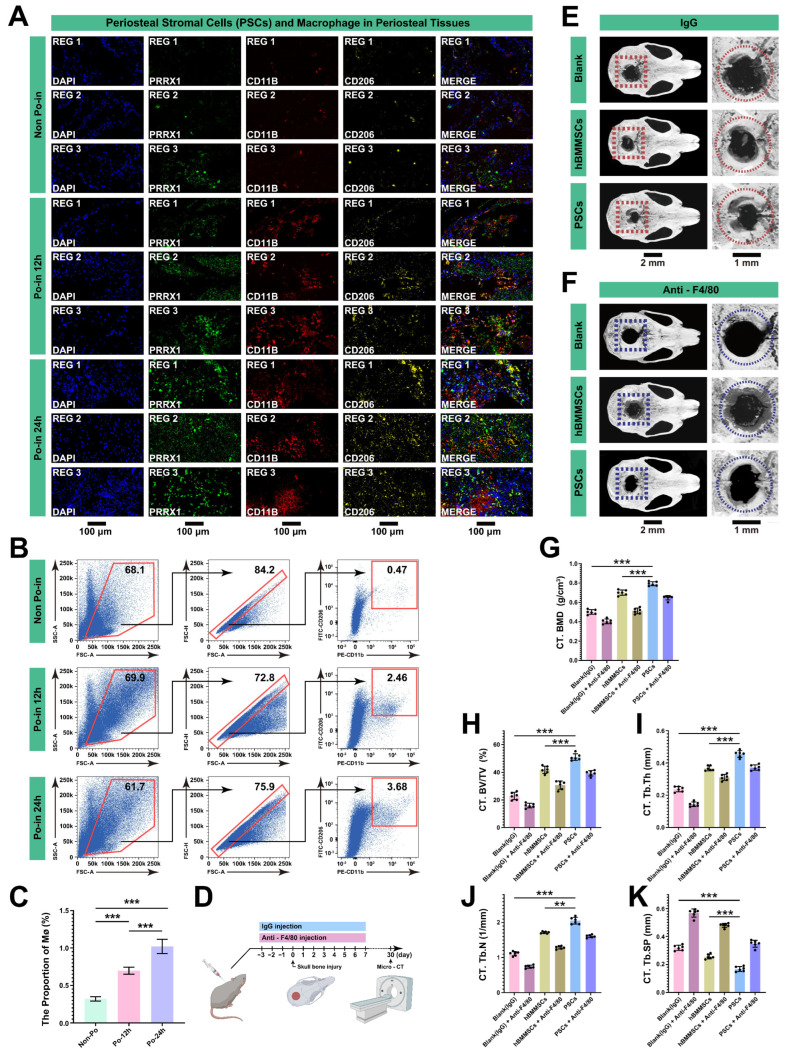
MØs are essential in the initial stages of bone reconstruction following cranial injury. (**A**) IHC clearly displays the locations of periosteal stem cells (PSCs) and MØs within three periosteal REGs under distinct injury conditions (DAPI, nucleus; Green, PRRX1; Red, CD11B; Yellow, CD206). Scale bar: 100 μm. (**B**) Representative FACS profiles of MØ populations under distinct injury conditions in miniature pigs. (**C**) Quantitative analysis of FACS profiles of MØs from (**D**) (*n* = 3, ***, *p* < 0.001). (**D**) Schematic representation of MØ consumption examination in critical cranial bone defects of C57/BL6 mice. (**E**,**F**) Micro-CT imaging of critical cranial bone defects in C57BL/6 mice recipients that received various cells. Dotted box indicates the cranial bone defect areas; dotted circle outlines the borehole boundary; scale bars: 2 and 1 mm. (**G**–**K**) Imaging parameter (BMD, BV/TV, Tb.Th, Tb.N, Tb.SP) analysis of the Micro-CT detection in (**G**) (*n* = 6, **, *p* < 0.01, ***, *p* < 0.001, black dots in the bar, the scattered distribution of data points).

Immunohistochemistry (IHC) clearly revealed the changes in these two cell types: the MØs were nearly absent in the periosteal tissue before the skull injury (all REGs) (Figure 5A); however, more MØs appeared in the periosteal tissue adjacent to the injury site (REG 1–3), with an increasing trend over time (Figure 5A). Similarly, we conducted FACS and an analysis of the MØs in the periosteal tissue of the three groups, finding that the cranial injury significantly stimulated the increase in the MØs in a time-dependent manner (Figure 5B,C).

To further confirm the regulatory role of MØs, we used neutralizing antibodies to evaluate their effect on bone repair in critical cranial bone defects of C57BL/6 mice. From three days before to seven days after creating cranial bone defects, MØs were depleted by the continuous injection of anti-F4/80 (a marker for mature mouse MØs), with the IgG antibody as a control. During this procedure, hBMMSCs and PSCs (Po-in 24 h group, carried by HAMA Gel) were transplanted into the bone defects of each group, with a comparative analysis also conducted with the control group (cell-free group). Micro-CT examinations were performed after a four-week healing period; imaging radiographs (Figure 5E,F) and an analysis of the serial bone morphological parameters (including the BMD, BV/TV, Tb.Th, Tb.N, and Tb.Sp) were conducted to evaluate the bone healing (Figure 5G–K). The results clearly showed that anti-F4/80 injection alone (cell-free) significantly blocked cranial healing, with virtually no new bone or osteoid formation in the cranial bone defects. Moreover, anti-F4/80 effectively reduced the contribution of both the hBMMSCs and PSCs to the bone formation, thereby prolonging the healing period. Additionally, we discovered that the PSCs had a superior bone repair efficiency compared to the hBMMSCs, even under the sole injection of IgG.

These morphological examinations indicate that the initial days post-injury are critical for early bone healing, and that the ablation of MØs not only impacts the migration and expansion of PSCs but also hinders the early bone-healing process in vivo, leading to delayed healing or non-healing. Therefore, we have reason to believe that MØs play a critical regulatory role in the migration, proliferation, and osteogenic function of cranial PSCs in the initial stages of post-bone injury.

### 3.6. Both M2DM and NRG1 Promote the Proliferation, Migration, and Osteogenic Activity of Cranial PSCs

**Figure 6:**
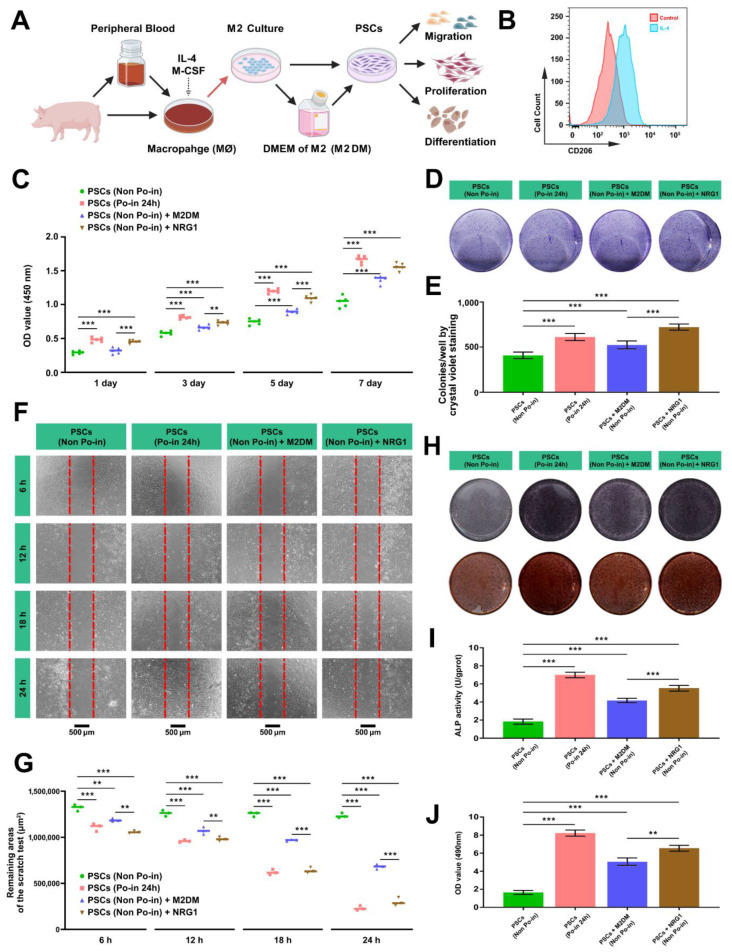
Both M2DM and NRG1 enhance the proliferation, migration, and osteogenic activity of cranial PSCs. (**A**) Schematic diagram showing the examination of the biological functions of the PSCs under the induction effects of MØs. (**B**) FACS identification of M2 macrophage cell counts after stimulation with IL-4. (**C**) Proliferation analysis of PSCs under various induction culture conditions at 1 d, 3 d, 5 d, and 7 d by CCK8 (*n* = 5, **, *p* < 0.01, ***, *p* < 0.001). (**D**) CFU detection of PSCs after 7 days under different induction culture conditions using crystal violet staining. (**E**) Quantitative analysis of CFU number of PSCs according to (**D**) (*n* = 3, ***, *p* < 0.001). (**F**) Scratch assay to assess morphology changes of PSCs at 6 h, 12 h, 18 h, and 24 h under different induction conditions. Scale bar: 500 μm. (**G**) Quantitative analysis of the remaining areas in scratch assay of PSCs (*n* = 3, **, *p* < 0.01, ***, *p* < 0.001). (**H**) ALP and ARS staining showing the in vitro osteogenic ability of PSCs under different induction culture conditions. (**I**,**J**) Quantitative analysis of ALP and ARS staining.

To more intuitively verify the recruiting effect of MØs on PSCs, we first isolated MØs from the peripheral blood of miniature pigs by gradient centrifugation [33], inducing M2-type polarization with Macrophage Colony-Stimulating Factor (M-CSF) and IL-4 [32] (Figure 6A). Using flow cytometry, M2 MØs were identified by CD206 (a classical macrophage polarization marker [32]) and cultured in vitro. Subsequently, the supernatant was collected for follow-up experiments (Figure 6B).

To evaluate the effect of the peripheral blood-derived MØs on the PSCs, we initially measured the proliferation activity. Given that NRG1 is a critical regulatory molecule in PSC crosstalk (Figure 4E), we also assessed the impact of recombinant NRG1 on the PSCs. CCK-8 assays confirmed that cranial injury promotes the proliferation of PSCs (Non Po-in vs. Po-in 24 h). Furthermore, both the M2DM and recombinant NRG1 enhanced the cell proliferation compared to the control cells alone (Figure 6C). Similarly, the colony-forming unit (CFU) ability of the PSCs, assessed by crystal violet staining, demonstrated that cranial injury enhanced the CFU formation in the PSCs, with a comparable promotional effect under the intervention of M2DM and recombinant NRG1 (Figure 6D,E). During a 6–24 h period, scratch assays revealed that the migration ability of the PSCs was superior following cranial injury, with the PSCs from non-injured skulls showing the slowest migration rate. Notably, the introduction of the M2 macrophage culture medium or NRG1 significantly accelerated the migration rate of the PSCs (Non Po-in), particularly in the NRG1 group (Figure 6F,G). Thus, both M2DM and NRG1 optimized the migration level of the PSCs. To our satisfaction, M2DM and NRG1 also enhanced the osteogenic differentiation and functional activity of the PSCs to varying degrees (Figure 6H–J).

Therefore, this section demonstrates the biological functions and activity changes of PSCs under cranial injury and provides evidence of the promotional effects of M2DM and NGR1 on the proliferation, migration, and osteogenic differentiation in PSCs.

## 4. Discussion

Bone tissue undergoes continuous remodeling throughout life, with the periosteum playing a pivotal role [34]. It extends beyond basic functions, regulating skeletal system homeostasis and facilitating adaptive remodeling [13,34]. Furthermore, the remarkable regenerative capabilities of PSCs are indispensable in the healing and reconstruction of bone following injuries [15,34]. Current studies on PDSCs primarily employ the Cre/LoxP system for genetic lineage tracing to elucidate their roles in bone regeneration. Research has identified various PSC populations, including PRRX1^+^ [35], platelet-derived growth factor receptor-alpha (PDGFR-α) [22], cathepsin K^+^(CTSK^+^) [10], alpha-smooth muscle actin (α-SMA^+^) [36], MX1^+^/α-SMA^+^ cells [21], and GLI1^+^ PDSCs [25]. Although the studies on PDSCs have shed light on their biological behavior, significant limitations remain. For instance, PDSCs derived from long bones may not accurately represent the functions of those from craniofacial tissues. Moreover, there are notable differences between the PSCs from rodent models and those from large animals and humans. Additionally, the field of immunology has scarcely been applied to the exploration of PSC characteristics.

Bone healing post-injury is a complex physiological process, involving various cell types and responses [37]. Initially, injury or trauma leads to the rupture of blood vessels and nerve fibers, triggering an acute inflammatory response within the first 24 h [15], which significantly compromises bone homeostasis. This intricate multicellular environment is vital in determining the activation and eventual fate of PSCs.

The inflammatory response initiates within the first few hours following bone injury as immune cells migrate from surrounding areas to the fracture site [38], leading to the infiltration of periosteal tissue. Evidence suggests that, at the onset of this process, MØ populations are present in both the germinal and fibrous layers of the periosteum [39]. More critically, extracellular matrix and periosteal stromal cells also participate in modulating the immune response by inhibiting the MØ polarization towards the M1 phenotype and promoting a shift towards the M2 type [40]. The polarization and activation of MØs are essential not only during the initial inflammatory phase of bone injury but also throughout the entire bone repair process, as the removal of MØs leads to the complete failure of bone regeneration in vivo [41]. Additionally, a shift in the MØ polarization from M1 to M2 can initiate an osteogenic response in PSCs, thereby facilitating bone reconstruction at the injury site [42]. Evidence also indicates that MØs can attract PSCs to the site and affect osteogenic differentiation by secreting PDGF-BB [43]. Beyond these findings, various perspectives have emerged on the regulatory effects of MØs on stem cells. For instance, some studies suggest that M1 and M2 polarizations of MØs play distinct roles at different stages of stem cell osteogenic differentiation: M1 polarization may enhance early differentiation but has no effect on the mineralization of the extracellular matrix, whereas only M2 MØs promote this mineralization [44]. Further research has shown that the ratio of MØs to stem cells at bone injury sites significantly influences the bone repair and reconstruction, with an optimal ratio of 5:1 necessary for matrix mineralization [45]. A recent study indicated that the MØs in the bone injury area were predominantly M2, with M1 nearly undetectable [46]. It is important to note that the single-cell RNA sequencing of swine periosteum cells suggests a dominant regulatory role for M2-type macrophages in PSCs during the early stages of the immune response following injury. However, given the differences between large animals and small rodents, alternative outcomes cannot be discounted. Thus, we used the classic F4/80 anti-mouse macrophage antibody for in vivo validation. Future studies will continue to explore macrophage subtypes during bone healing in mice.

NRG1 is a critical epidermal growth factor (EGF) family ligand that plays an important role in the tissue repair process after injury [47]. Moreover, evidence depicts that the *NRG1* expression levels in macrophages are elevated significantly after injury compared to the homeostatic conditions, as well as the stromal cells [47]. In this study, the specific influences of the M2 MØs on PSCs were investigated, and we also explored the specific function of NRG1 separately. Compared with M2DM, we found that NRG1 can significantly enhance the proliferation, cloning, migration, and osteogenic differentiation activities of PSCs. Our results are consistent with the above studies, and they also implicate the specific role of NRG1 in periosteal stromal cells in response to tissue injury.

To summarize, the initial phase of bone injury is a critical period for the inflammatory response, involving differentiations and interactions among various cell types. During these pivotal stages, the specific roles of stem cells within the periosteum, particularly those from craniofacial tissues, remain poorly defined. Additionally, the regulatory effects of M1 and M2 MØs on the osteogenic differentiation of PSCs during tissue regeneration, both temporally and spatially, are not yet fully understood. In this study, we selected miniature pigs, which exhibit anatomical and developmental characteristics more closely aligned with those of humans, as our animal model. The study focused on exploring PSCs during the early stages following cranial bone injury (within 24 h) and investigated the interactions between MØs and PSCs using single-cell sequencing technology. We conclusively demonstrated the role of M2-type MØs in enhancing the proliferation, migration, and osteogenesis of PSCs through comprehensive in vivo and in vitro analyses. This research not only elucidates the differentiation and functional regulation of stem cells derived from craniomaxillofacial tissues from an immunological perspective but also establishes a theoretical foundation for the reconstruction of craniomaxillofacial bone injuries in translational medicine. Further studies are required to define the specific identities of particular cells, while the molecular regulatory mechanisms between PSCs and MØs also warrant further investigation and discussion.

## 5. Conclusions

In our study, we established a miniature pig model of cranial bone injury and observed that M2 macrophages guide the recruitment of PSCs and initiate bone regeneration. This finding not only deepens our understanding of the periosteum’s immune microenvironment but also suggests new directions and potential therapeutic targets for immunoregulatory strategies in cranial bone injury repair. By precisely modulating this process, future treatments could be developed to enhance bone repair and regeneration.

## Data Availability

The single-cell RNA sequencing (scRNA-seq) datasets of the miniature pigs generated in this study are publicly available at the China National Center for Bioinformation (CNCB). These datasets can be accessed via https://ngdc.cncb.ac.cn/gsa/ under accession numbers PRJCA023544.
